# Prevalence of *Escherichia coli* ST1193 Causing Intracranial Infection in Changsha, China

**DOI:** 10.3390/tropicalmed7090217

**Published:** 2022-08-31

**Authors:** Yi-Ming Zhong, Xiao-He Zhang, Zheng Ma, Wen-En Liu

**Affiliations:** 1 Department of Clinical Laboratory, Xiangya Hospital, Central South University, Changsha 410008, China; 2 National Clinical Research Center for Geriatric Disorders, Xiangya Hospital, Central South University, Changsha 410008, China; 3 Faculty of Laboratory Medicine, Xiangya School of Medicine, Central South University, Changsha 410013, China

**Keywords:** *E. coli* ST1193, intracranial infections, antibiotic resistant, meningitis, clinical characteristics

## Abstract

ST1193 is an emerging new virulent and resistant clone among *Escherichia coli* with a tendency to spread rapidly across the globe. However, the prevalence of intracranial infection-causing *E. coli* ST1193 is rarely reported. This study aimed at determining the prevalence of *E. coli* ST1193 isolates, causing intracranial infections in Changsha, central China. A total of 28 *E. coli* isolates were collected from the cerebrospinal fluid of patients with intracranial infection over a four-year period. All isolates were differentiated using multilocus sequence typing (MLST), and phylogenetic grouping, and tested for antibiotic resistance. MLST analysis showed 11 sequence types (ST) among the 28 *E. coli* isolates. The most prevalent ST was B2-ST1193 (28.6%, 8/28), followed by B2-ST131 (21.4%, 6/28) and F-ST648 (10.7%, 3/28). Of the eight ST1193 isolates, three carried CTX-M-55, and one carried CTX-M-27. All eight ST1193 isolates were resistant to Ciprofloxacin, showing *gyrA*1AB/*parC*4A mutations. Two ST1193 isolates carried the *aac(6′)-Ib-cr* gene. All ST1193 isolates were recovered from infants with meningitis, with a fatal outcome for one three-month-old infant. ST1193 has emerged as the predominant type of *E. coli* strain causing intracranial infections in Changsha, China. This study highlights the importance of implementing appropriate surveillance measures to prevent the spread of this emerging public health threat.

## 1. Introduction

*Escherichia coli* is usually considered to be a part of the normal intestinal flora of humans. However, extraintestinal pathogenic *E. coli* (ExPEC) can cause a range of diseases, including sepsis, meningitis, and urinary tract infections [1,2,3]. *E. coli* is a major cause of neonatal bacterial meningitis and is responsible for approximately 33% of the cases [4]. Although less common, *E. coli* can also cause meningitis in adults [5]. Neonatal meningitis caused by *E. coli* is associated with fatality rates ranging from 5 to 30% in infants and a high incidence of neurological sequelae [6,7,8].

ST1193 is emerging as a new virulent clone among fluoroquinolone-resistant *E. coli* in several countries [9,10,11,12,13]. Most studies detected this lineage as a dominant clinical *E. coli* isolate, collected from urine and bloodstream samples [10,11,12]. Few studies have focused on the prevalence of this ST in cerebrospinal fluid [14]. A recent Chinese study reported that ST1193 was the most frequent sequence type in neonatal invasive *E. coli* infections, but not limited to cerebrospinal fluid [14]. Thus, whether ST1193 was also the predominant clone in cerebrospinal fluid is still uncertain. Recently, Oldendorff et al. reported a case in which ST1193 infection in a preterm newborn with meningitis led to a fatal outcome [15]. Therefore, more studies are warranted to broaden our knowledge on ST1193 causing intracranial infections.

In this study, we aim to determine the prevalence of ST1193 isolates causing intracranial infections collected from the cerebrospinal fluid of patients with intracranial infections over the period of four years in a large university-affiliated hospital in China. The clinical characteristics and outcomes of infections caused by *E. coli* ST1193 were also assessed.

## 2. Materials and Methods

### 2.1. Bacterial Isolates

From January 2013 to December 2016, a total of 28 non-duplicate *E. coli* clinical isolates were consecutively collected from cerebrospinal fluid from patients with intracranial infections at a general teaching hospital affiliated with Central South University (Changsha, Hunan Province, China). The 28 samples were collected as a routine sampling procedure and represent the total number of these types of infections over a four-year period. The diagnosis of intracranial infection and meningitis were defined according to established diagnostic criteria [16,17]. This study was performed in line with the principles of the Declaration of Helsinki. Approval was granted by the Ethics Committee of the Xiangya Hospital of Central South University (reference number 202112178).

### 2.2. Multilocus Sequence Typing and Phylogenetic Group

Multilocus sequence typing (MLST) was performed on all isolates. Primers and PCR conditions for the seven housekeeping genes commonly used in *E. coli* MLST schemes (*adk*, *fumC*, *icd*, *mdh*, *purA*, *recA*, and *gyrB*) were obtained from databases at the University of Warwick (http://mlst.warwick.ac.uk/mlst/dbs/Ecoli) (accessed on 4 August 2022). The sequences of primers and annealing temperature were presented in Supplementary Table S1. Sequence data were analyzed using the *E. coli* MLST database. The phylogenetic groups of *E. coli* were determined by quadruplex PCR [18].

### 2.3. Antimicrobial Susceptibility Testing

Susceptibility testing was performed using the Vitek 2 system (bioMérieux, Marcy-l’Étoile, France). Susceptibilities to the following drugs were determined: ampicillin, cefazolin, ceftriaxone, ampicillin/sulbactam, piperacillin/tazobactam, imipenem, gentamicin, ciprofloxacin, aztreonam, trimethoprim/sulfamethoxazole. The results were interpreted according to Clinical and Laboratory Standards Institute (CLSI) criteria [19]. Extended-spectrum β-lactamase (ESBL) production was confirmed using the double disc synergy test. The *E. coli* ATCC 25922 strain was used as standard quality control. The intermediate susceptibility of isolates towards antibiotics was considered resistant.

### 2.4. Molecular Characterization of ST1193

For ST1193 isolates, detection of *bla*_CTX-M_ was performed using PCR and amplicon sequencing [20,21]. The quinolone-resistance determining regions (QRDRs) in *gyrA* and *parC* genes were identified and amplicon sequencing was performed as previously described [22]. The screening of plasmid-mediated quinolone resistance (PMQR) determinants [*qnrA*, *qnrB*, *qnrC*, *qnrS*, *aac(6′)-Ib-cr*, and *qepA*] was also conducted using PCR [23]. The sequences of primers for PCR amplification and annealing temperature are shown in Supplementary Table S1. The amplified PCR products were sequenced, and the results were analyzed by comparing them to sequences in GenBank (https://blast.ncbi.nlm.nih.gov/Blast.cgi) (accessed on 4 August 2022).

### 2.5. Clinical Data Collection

We reviewed the medical records of the patients with ST1193 infection and collected patient data, including data on demographic characteristics, clinical manifestations, laboratory tests, treatments, and clinical outcomes.

### 2.6. Statistical Analysis

Comparisons of proportions between groups were assessed via Fisher’s exact test. A value of *p* < 0.05 was considered to be statistically significant.

## 3. Results

### 3.1. Multilocus Sequence Typing and Phylogenetic Group

MLST analysis revealed a total of 11 STs in the 28 *E. coli* isolates. Table 1 presents the distribution of ST types among the 28 *E. coli* isolates. The main STs were ST1193, ST131, and ST648, accounting for 28.6% (8/28), 21.4% (6/28), and 10.7% (3/28), respectively. The other types were ST354, ST38, and ST457, each ST accounting for 7.1% (2/28); ST58, ST69, ST23, ST95, and ST394, each ST accounting for 3.6% (1/28).

Phylogenetic analysis of the 28 *E. coli* strains showed that 15 (53.6%) belonged to phylogroup B2, 7 (25.0%) to phylogroup F, 3 (10.7%) to phylogroup D, 1 (3.6%) to phylogroup B1, 1 (3.6%) to phylogroup C, and 1 (3.6%) to phylogroup E.

### 3.2. Antimicrobial Susceptibility

Of the 28 isolates, 26 (92.9%) were found to be resistant to ampicillin, 22 (78.6%) to trimethoprim/sulfamethoxazole and cefazolin, 21 (75%) to ampicillin/sulbactam, and 19 (67.9%) to ceftriaxone and gentamicin (Table 2). Out of the 28 isolates, 60.7% (17/28) were ESBL producers.

As shown in Table 2, the ST1193 isolates had a significantly higher prevalence of resistance to ciprofloxacin compared with non-ST1193. Ciprofloxacin resistance was found in 100% of the ST1193 isolates, whereas 45% of the non-ST1193 isolates were resistant.

### 3.3. Molecular Characterization of ST1193

Four of the eight ST1193 strains (50%) were recognized as ESBL producers. Three ESBL-producing isolates carried CTX-M-55, and the remaining carried CTX-M-27. All eight ciprofloxacin-resistant ST1193 isolates possessed a set of three amino acid replacement mutations (*gyrA*1AB Ser-83-Leu, Asp-87-Asn, and *parC*4A Ser-80-Ile) in QRDRs. Two ST1193 isolates carried the *aac(6′)-Ib-cr* gene. None of the other types of PMQR determinants was detected in the tested isolates.

### 3.4. Clinical Characteristics of the Patients with ST1193 Infection

The demographical and clinical parameters of the eight patients with intracranial infections carrying ST1193 isolate are summarized in Table 3. All ST1193 isolates were recovered from infants with meningitis. They all exhibited typical manifestations, and their laboratory test results supported their diagnosis. Meropenem treatment was provided to all the selected patients; wherein, all except one patient showed recovery, and one had a fatal outcome. The patient with a fatal outcome was a three-month-old female exhibiting the symptoms of fever, neonatal coma, and convulsion. She was diagnosed with meningitis accompanying subdural effusion, hydrocephalus, and cerebral hernia. The disease progressed rapidly and led to an eventual fatality.

## 4. Discussion

ST1193 is an emerging new virulent and resistant clone among fluoroquinolone-resistant *E. coli* with a tendency to spread rapidly across the globe. Initial studies on ST1193 reported their isolation mostly from adult urine and a few from blood samples [10,11,12]. Ding et al. initially detected ST1193 isolate in neonatal blood and cerebrospinal fluid specimens of patients with meningitis [14]. Although the study provided the first evidence for the involvement of ST1193 in intracranial infections, it did not provide evidence for its predominance over other isolates in the cerebrospinal fluid of meningitis patients [14]. In the present study, we focused on determining the predominant ST of *E. coli* isolated from CSF of patients with intracranial infections. This is the first study to provide evidence to substantiate the prevalence and predominance of ST1193 (28.6%) over other STs in CSF of patients with intracranial infections. Berman et al. reported that the STs of *E. coli* isolates that were involved in causing meningitis included, ST131, ST69, ST405, and ST62, with no evidence for the detection of ST1193 [24]. These disparities may be due to geographical or host population differences. This finding implies that ST1193 may be poised to emerge as a major type of *E. coli* in patients with intracranial infections in China. A recent review described that ST1193 is following in the footsteps of the most successful MDR *E. coli* clone named ST131 [9]. Our study demonstrated that ST1193 has surpassed ST131 in *E. coli* intracranial infections in Changsha, China, which means this clone has become a public health threat and should be a concern. In order to investigate the emergence of ST1193, basic characteristics, surveillance, and clinical studies must be conducted on an urgent basis [9]. The information will be useful for managing and preventing this infectious disease.

Previous studies have reported that CTX-M-14 is the most prevalent ESBL genotype in China [25,26,27]. Wu et al. described that the CTX-M-14 genotype accounted for more than 50% of the *bla*_CTX-M_ positive ST1193 isolates [28]. However, in the present study, CTX-M-55 (a single locus variant (SLV) of CTX-M-15) was a more popular genotype among the ESBL-producing ST1193 isolates. A previous study speculated that dissemination of the *bla*_CTX-M-55_ in China may be partly because of the widespread prevalence of the *E. coli* ST1193 clone [29]. It has been reported in recent years that the worldwide increase in *E.coli-*producing CTXM-15 enzymes has been linked to epidemic clone ST131 [30]. CTX-M-55 has higher hydrolytic activity than CTX-M-15 and increased catalytic efficiency against ceftazidime and cefotaxime [31,32]. In this study, however, there was not enough data to determine whether CTX-M-55 was related to ST1193 due to the small sample size. Therefore, more studies with larger sample sizes are required to determine a putative association between ST1193 and the presence of CTX-M-55.

Consistent with the findings of Ding et al. [14], we found that all the ST1193 isolates were resistant to ciprofloxacin. The emergence of fluoroquinolone resistance of *E. coli* may be caused as a result of precise, analogous point mutations within the QRDRs of GyrA and ParC, the fluoroquinolone targets [22]. In the present study, all ciprofloxacin-resistance ST1193 isolates harbored the same distinctive mutations of three amino acid substitutions (*gyrA*1AB Ser-83-Leu, Asp-87-Asn, and *parC*4A Ser-80-Ile), complying with the previous reports [12,28,33]. The homogeneity of the ST1193 isolates suggests that this clone probably derived and spread from a common ancestor [12,28]. Johnson et al. reported that distinctive mutations in *gyrA* and *parC* may confer a fitness advantage for ST131 *H*30 isolates over non-*H*30 fluoroquinolone-resistant isolates [34]. Similarly, the particular *gyrA/parC* mutations of ST1193 may give this clone an advantage and promote its spread. Further research is necessary to verify this speculation and elucidate the role of these *gyrA/parC* mutations in ST1193. It seems that ST1193 isolates have some common features such as fluoroquinolone resistance, and QRDR mutations [9]. A high degree of homogeneity between ST1193 strains from different infection sites and geographical locations suggests they are likely descendants of the same ancestor [28]. Further studies are required to elucidate and monitor the evolution of ST1193.

In this study, all ST1193 isolates were collected from infants with meningitis. Although neonatal meningitis is an important cause of neonatal mortality, only a few studies have reported ST1193 infection in this fatal disease [14,15,35]. Ding et al. reported that ST1193 was the most common and invasive neonatal *E. coli* clone recovered from blood and CSF [14]. Only two recent case reports have described the fatal cases of neonatal meningitis caused by *E. coli* ST1193, one each in America and Sweden [15,35]. In our study, one patient who had a fatal outcome also suffered a similar fulminant course as that described in previous studies [15,35]. Therefore, considering the potential of this isolate in rapidly emerging, spreading, and causing severe outcomes, it requires adequate attention and implementation of appropriate measures to prevent its spread, especially in neonatal meningitis.

One limitation of this study was the small number of *E. coli* isolates due to the precious specimen source. Thus, a multicenter study with larger sample sizes is needed to further explore the characterization of ST1193 in CSF.

## 5. Conclusions

In summary, ST1193 has emerged as the predominant type of *E. coli* strain causing intracranial infections in patients in Changsha, China. It is an important public health issue, especially since this clone became the dominant strain of *E. coli* that caused this infectious disease. Considering the virulence and multidrug resistance, effective surveillance should be implemented to prevent the spread of ST1193 isolates in patients with intracranial infections.

## Figures and Tables

**Table 1:** Distribution of ST types and phylogenetic groups among the 28 *E. coli* isolates.

MLST	Allelic Profile							Phylogenetic Group	No. (%) of Strains
	*adk*	*fumC*	*gyrB*	*icd*	*mdh*	*purA*	*recA*		
ST1193	14	14	10	200	17	7	10	B2	8 (28.6)
ST131	53	40	47	13	36	28	29	B2	6 (21.4)
ST648	92	4	87	96	70	58	2	F	3 (10.7)
ST354	85	88	78	29	59	58	62	F	2 (7.1)
ST38	4	26	2	25	5	5	19	D	2 (7.1)
ST457	101	88	97	108	26	79	2	F	2 (7.1)
ST58	6	4	4	16	24	8	14	B1	1 (3.6)
ST69	21	35	27	6	5	5	4	D	1 (3.6)
ST23	6	4	12	1	20	13	7	C	1 (3.6)
ST95	37	38	19	37	17	11	26	B2	1 (3.6)
ST394	21	35	61	52	5	5	4	E	1 (3.6)

MLST: multi-locus sequence typing; ST: sequence type.

**Table 2:** Antimicrobial resistance of the 28 *E. coli* isolates.

Antimicrobial Resistance	No. (%) of Strains (n = 28)	No. (%) of Strains		*p*-Value *
		ST1193 (n = 8)	Non-ST1193 (n = 20)	
Ampicillin	26 (92.9)	7 (87.5)	19 (95.0)	0.497
Cefazolin	22 (78.6)	5 (62.5)	17 (85.0)	0.311
Ceftriaxone	19 (67.9)	4 (50.0)	15 (75.0)	0.371
Ampicillin/sulbactam	21 (75.0)	6 (75.0)	15 (75.0)	1.000
Piperacillin/tazobactam	1 (3.6)	1 (12.5)	0 (0)	0.286
Imipenem	1 (3.6)	0 (0)	1 (5.0)	1.000
Gentamicin	19 (67.9)	5 (62.5)	14 (70.0)	1.000
Ciprofloxacin	17 (60.7)	8 (100)	9 (45.0)	0.010
Aztreonam	12 (42.9)	3 (37.5)	9 (45.0)	1.000
Trimethoprim/sulfamethoxazole	22 (78.6)	7 (87.5)	15 (75.0)	0.640
ESBLs	17 (60.7)	4 (50.0)	13 (65.0)	0.671

* ST1193 versus non-ST1193.

**Table 3:** Clinical characteristics and CTX-M genotype status in the eight patients with intracranial infections carrying ST1193 isolate.

Clinical Characteristics	Patient 1	Patient 2	Patient 3	Patient 4	Patient 5	Patient 6	Patient 7	Patient 8
Age (days)	85	26	12	61	6	92	13	79
Gender	Male	Female	Male	Female	Female	Female	Female	Female
Fever	+	+	+	+	+	+	+	+
Convulsion	-	-	+	-	-	+	-	-
Meningitis	+	+	+	+	+	+	+	+
Subdural effusion	-	+	-	-	-	+	-	+
Laboratory test (CSF)								
WBC (×10^6^/L)	7250	1280	2220	1020	2100	12820	2900	1310
Glucose (mmol/L)	0.20	0.43	0.24	0.25	0.71	0.06	0.02	0.14
Protein (mg/L)	2880	2140	2660	3370	2370	3520	2840	3010
Treatment	MEM, TZP	MEM, CRO, AMC	MEM, SAM, CRO	MEM	MEM, CRO, AMC	MEM, CRO	MEM	MEM, CRO
Length of stay (days)	38	30	56	26	29	8	38	43
Outcome	Survived	Survived	Survived	Survived	Survived	Death	Survived	Survived
CTX-M genotype	CTX-M-27	-	-	CTX-M-55	-	CTX-M-55	CTX-M-55	-

CSF: cerebrospinal fluid; MEM: meropenem; TZP: piperacillin/tazobactam; CRO: ceftriaxone; AMC: amoxicillin/clavulanic acid; SAM: ampicillin/sulbactam.

## Data Availability

The data that support the findings of this study are available from the corresponding author (W.-E.L.) on reasonable request.

## Supplementary Materials

The following supporting information can be downloaded at: https://www.mdpi.com/article/10.3390/tropicalmed7090217/s1, Table S1: Sequences of primers for PCR amplification and annealing temperature.
