# Supplementary material for: Prevalence of Escherichia coli ST1193 Causing Intracranial Infection in Changsha, China
Source: Trop Med Infect Dis. 2022 Aug 31;7(9):217. doi: 10.3390/tropicalmed7090217 (PMC9504535; doi:10.3390/tropicalmed7090217)
Supplement: Supplementary file 1 [file tropicalmed-07-00217-s001.zip › tropicalmed-1875457-supplementary.pdf]

Tropical Medicine and Infectious Disease

MDPI

Supplementary Materials: Prevalence of *Escherichia coli* ST1193 causing intracranial infection in Changsha, China

Table S1. Sequences of primers for PCR amplification and annealing temperature.

Gene Name	Primer	Primer Sequences (5′–3′)	Primer Size (bp)	Annealing Temperature/◦C
adk	Forward	ATTCTGCTTGGCGCTCCGGG	583	54
	Reverse	CCGTCAACTTTCGCGTATTT		
	Forward1	TCATCATCTGCACTTTCCGC		
	Reverse1	CCAGATCAGCGCGAACTTCA		
fumC	Forward	TCACAGGTCGCCAGCGCTTC	806	54
	Reverse	GTACGCAGCGAAAAAGATTC		
	Reverse1	TCCCGGCAGATAAGCTGTGG		
gyrB	Forward	TCGGCGACACGGATGACGGC	911	60
	Reverse	ATCAGGCCTTCACGCGCATC		
	Reverse1	GTCCATGTAGGCGTTCAGGG		
icd	Forward	ATGGAAAGTAAAGTAGTTGTTCCGGCAC	878	54
	Reverse	GGACGCAGCAGGATCTGTT		
mdh	Forward	ATGAAAGTCGCAGTCCTCGGCGCTGCTGGCGG	932	60
	Reverse	TTAACGAACTCCTGCCCCAGAGCGATATCTTTCT T		
	Forward1	AGCGCGTTCTGTTCAAATGC		
	Reverse1	CAGGTTCAGAACTCTCTCTGT		
purA	Forward	CGCGCTGATGAAAGAGATGA	816	54
	Forward1	TCGGTAACGGTGTTGTGCTG		
	Reverse	CATACGGTAAGCCACGCAGA		
recA	Forward	CGCATTCGCTTTACCCTGACC	780	58
	Reverse	TCGTCGAAATCTACGGACCGGA		
	Forward1	ACCTTTGTAGCTGTACCACG		
	Reverse1	AGCGTGAAGGTAAAACCTGTG		
chuA	Forward	ATGGTACCGGACGAACCAAC	288	59
	Reverse	TGCCGCCAGTACCAAAGACA		
yjaA	Forward	CAAACGTGAAGTGTCAGGAG	211	59
	Reverse	AATGCGTTCCTCAACCTGTG		
TspE4.C2	Forward	CACTATTCGTAAGGTCATCC	152	59
	Reverse	AGTTTATCGCTGCGGGTCGC		
arpA	Forward	AACGCTATTCGCCAGCTTGC	400	59
	Reverse	TCTCCCCATACCGTACGCTA		
ArpAgpE	Forward	GATTCCATCTTGTCAAAATATGCC	301	57
	Reverse	GAAAAGAAAAAGAATTCCCAAGAG		
trpAgpC	Forward	AGTTTTATGCCCAGTGCGAG	219	59
	Reverse	TCTGCGCCGGTCACGCCC		
trpBA	Forward	CGGCGATAAAGACATCTTCAC	489	
	Reverse	GCAACGCGGCCTGGCGGAAG		
bla$_{CTX-Mgroup1}$	Forward	AAAAATCACTGCGCCAGTTC	415	52
	Reverse	AGCTTATTCATCGCCACGTT		
	Forward1	CGTCACGCTGTTGTTAGGAA	780	55
	Reverse1	ACGGCTTTCTGCCTTAGGTT		
bla$_{CTX-Mgroup2}$	Forward	CGACGCTACCCCTGCTATT	552	52
	Reverse	CCAGCGTCAGATTTTTCAGG		
bla$_{CTX-Mgroup8}$	Forward	TCGCGTTAAGCGGATGATGC	666	52
	Reverse	AACCCACGATGTGGGTAGC		
bla$_{CTX-Mgroup9}$	Forward	CAAAGAGAGTGCAACGGATG	205	52
	Reverse	ATTGGAAAGCGTTCATCACC		
	Forward1	TATTGGGAGTTTGAGATGGT	932	50
	Reverse1	TCCTTCAACTCAGCAAAAGT		
bla$_{CTX-Mgroup25}$	Forward	GCACGATGACATTCGGG	327	52
	Reverse	AACCCACGATGTGGGTAGC		
gyrA	Forward	CGACCTTGCGAGAGAAAT	625	58
	Reverse	GTTCCATCAGCCCTTCAA		
parC	Forward	CGATTGCCGCCTGAGCCACTT	605	58
	Reverse	GCGAATAAGTTGAGGAATCAG		
qnrA	Forward	ATTTCTCACGCCAGGATTTG	516	53

	Reverse	GATCGGCAAAGGTTAGGTCA		
qnrB	Forward	GATCGTGAAAGCCAGAAAGG	476	53
	Reverse	ATGAGCAACGATGCCTGGTA		
qnrC	Forward	GGGTTGTACATTTATTGAATCG	307	53
	Reverse	CACCTACCCATTTATTTTCA		
qnrS	Forward	GCAAGTTCATTGAACAGGGT	428	53
	Reverse	TCTAAACCGTCGAGTTCGGCG		
aac(6)-Ib-cr	Forward	TTGCGATGCTCTATGAGTGGCTA	482	55
	Reverse	CTCGAATGCCTGGCGTGTTT		
qepA	Forward	AACTGCTTGAGCCCGTAGAT	596	55
	Reverse	GTCTACGCCATGGACCTCAC		
